# Successful management of a mediastinum abscess with sternum destruction caused by MSSA bloodstream infection

**DOI:** 10.1186/s40792-022-01440-7

**Published:** 2022-05-03

**Authors:** Hironobu Wada, Yuki Shina, Toshiko Kamata, Fumihiro Ishibashi, Hajime Tamura, Masahiro Toriumi, Kyoichi Matsuzaki, Shigetoshi Yoshida

**Affiliations:** 1 Department of Thoracic Surgery, International University of Health and Welfare, 852, Hatakeda, Narita, Chiba 286-8520 Japan; 2 Department of Plastic and Reconstructive Surgery, International University of Health and Welfare, 852, Hatakeda, Narita, Chiba 286-8520 Japan

**Keywords:** Multiple deep organ abscesses, MSSA bloodstream infection, Sternal resection

## Abstract

**Background:**

Multiple deep organ abscesses associated with *Staphylococcus aureus* bloodstream infection (SAB) have a high mortality rate, requiring rapid removal or drainage of infective foci with long-term appropriate antimicrobial therapy. Cases in which infective foci cannot be completely removed are challenging for their management.

**Case presentation:**

A 77-year-old man developed multiple deep organ abscesses associated with SAB. The left anterior chest subcutaneous abscess continued into the right anterior mediastinum and had extensively destroyed the sternum. Necrotizing fasciitis was observed in the bilateral feet. The anterior mediastinum abscess was drained percutaneously, and the chest wall abscess was incised cautiously without causing an external pneumothorax. On the next day, right-sided pyothorax had developed, requiring pleural drainage. On the third day, debridement of anterior chest wall abscess followed by concurrent thoracoscopic pleural curettage and debridement of bilateral feet were performed. Thorough sternal debridement was not performed, considering the risk of respiratory failure due to the sternal defects. On the 24th day, sternum debridement and incisional drainage of sciatic rectus fossa abscess, which had been present since the time of admission, were performed to control persistent infection. The caudal half of the sternal body was resected, leaving the costal cartilage attachments. The general condition further improved without postoperative respiratory failure after the second surgery, leading to a transfer to the general ward on the 43rd day.

**Conclusions:**

We successfully treated the severe multiple deep organ abscesses, including a mediastinum abscess with sternum destruction, by repeated removal of the infective foci while avoiding respiratory failure due to excessive debridement of the anterior chest wall, including the sternum.

## Background

*Staphylococcus aureus* is a common pathogen causing bloodstream infection, and *S. aureus* bloodstream infection (SAB) is reported to be associated with high morbidity and a 20% or higher mortality rate [1]. SAB can cause deep organ abscesses which have a 12-week mortality rate exceeding 30% and require prompt treatment [2]. Herein, we report a case of multiple deep organ abscesses, including a mediastinum abscess with sternum destruction, associated with methicillin-sensitive SAB that was successfully treated with multi-stage surgical drainage with appropriate antimicrobial therapy.

## Case presentation

**Fig. 1:**
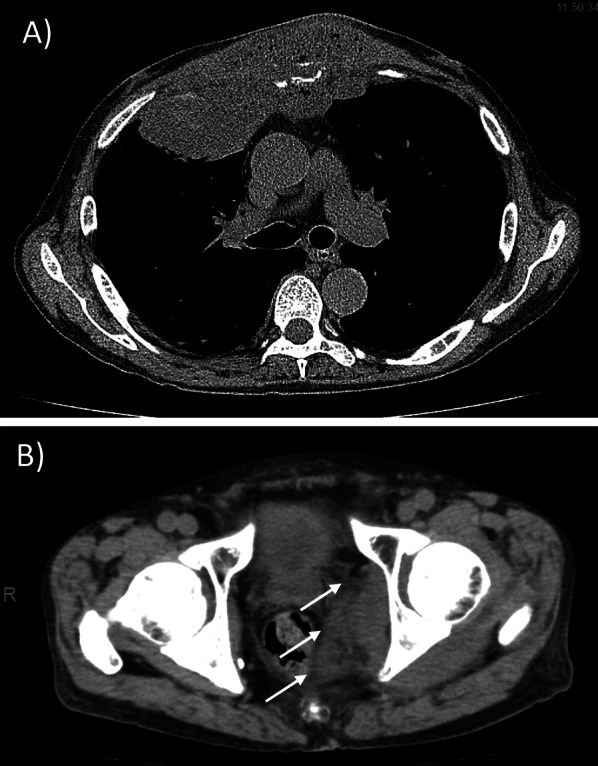
Computed tomography scans revealing multiple deep organ abscesses. A whole-body computed tomography (CT) scan showing an extensive abscess from the left subcutaneous into the right anterior mediastinum (**A**) and a sciatic rectus fossa abscess (**B**)

A 77-year-old man presented to a dermatology clinic with chief complaints of pain and swelling in the bilateral dorsum of his feet for 1 week. His past history included alcoholic liver disease. He was diagnosed with cellulitis of the bilateral feet and underwent incisional drainage at a municipal hospital. He also had a bulge in the anterior chest wall, where he had been hit forcefully 2 weeks previously. His whole-body computed tomography (CT) scan revealed an extensive abscess that had destroyed sternal body and continued from the left anterior chest to the right anterior mediastinum (Fig. 1). Therefore, he was promptly transferred to our department for the management of the thoracic lesion. On arrival, he was conscious, and his circulatory and respiratory dynamics were barely maintained with supplemental fluids and oxygenation at 2 L/min. Renal function was impaired with a creatinine level of 3.1 mg/dL. His anterior chest showed a 5-cm-diameter bulge with redness. Both the patient’s feet had necrotic fascia, suggesting necrotizing fasciitis rather than cellulitis. His whole-body CT confirmed not only a continuous abscess in the thorax, but also a left sciatic rectus fossa abscess in the pelvis (Fig. 1). Trans-thoracic echocardiography revealed no evidence of infective endocarditis. The diagnosis was multiple deep organ abscesses, including anterior chest wall abscess, anterior mediastinal abscess, sternal osteomyelitis, sciatic rectus fossa abscess, and necrotizing fasciitis of both feet. His APACHE II score was calculated to be 26, with a high predictive in-hospital mortality rate.

**Fig. 2:**
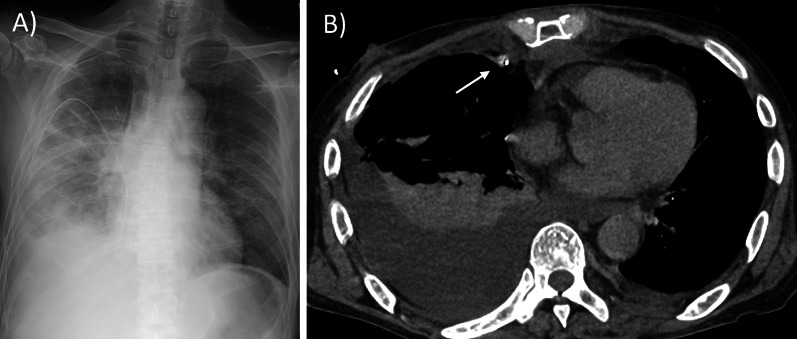
The right pleural effusion developed after mediastinal drainage. While a drain is inserted into the anterior mediastinum and the mediastinal abscess is reduced, a right massive pleural effusion occurred on the second day of admission (**A**, **B**). A white arrow shows the drainage tube (**B**)

Given that his general condition was unfavorable, and the right anterior mediastinum abscess was accessible, percutaneous drainage under local anesthesia was first performed in the emergency room via the right second intercostal space. Furthermore, small incisional drainage was performed for the left anterior chest wall abscess, and the necrotic pectoral muscles were debrided cautiously not to cause external pneumothorax via the abscess. After admission to the intensive care unit, vancomycin administration was initiated, which was subsequently switched to cefazolin when methicillin susceptible *S. aureus* was detected in blood and drained abscess culture tests. On the following day, while the mediastinal abscess shrunk, the right pleural effusion increased rapidly, likely due to mediastinal pleural injury during the first anterior mediastinal drainage (Fig. 2). Right pleural drainage showed pyothorax. The pleural effusion became more purulent rapidly over time after drainage. On the 3rd day of hospitalization, additional debridement of the anterior chest wall abscess and necrotizing fasciitis in both feet, and thoracoscopic pleural curettage were performed under general anesthesia as the systemic inflammation was not sufficiently improved by the previous drainage. The patient was positioned supine with a pillow under his back on the right side, and debridement of the anterior chest was performed, followed by concurrent thoracoscopic pleural curettage by thoracic surgeons and debridement of the lower extremities by plastic surgeons. Thoracoscopic pleural curettage was performed to control rapidly progressive pleural infection. The necrotic right second toe and left fifth toe were amputated. Although the sternal body was infected and extensively destroyed by the abscess, the sternum was left intact to prevent postoperative respiratory failure caused by sternal defects.

**Fig. 3:**
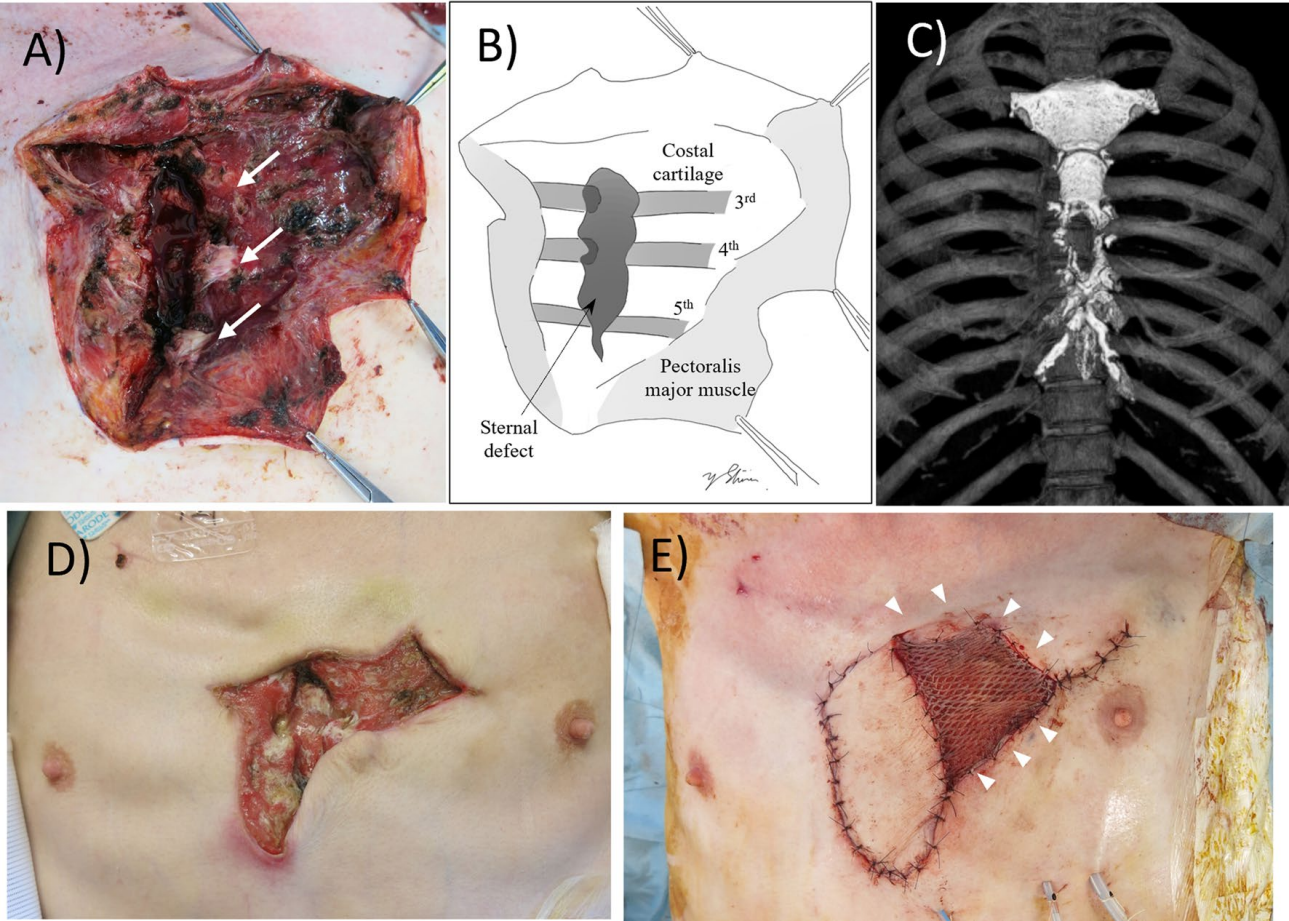
The sternal resection followed by latissimus dorsi myocutaneous flap reconstruction. **A**, **B** The anterior chest wall was widely incised, and the caudal half of the sternal body was resected leaving the costal cartilage attachments. The arrows show third, fourth, and fifth costal cartilage from the top. Light gray area shows remaining pectoralis major muscle, which was widely debrided. **C** Postoperative CT image showing sternal defect. **D**, **E** The anterior chest wall wound before (**D**) and after (**E**) latissimus dorsi myocutaneous flap reconstruction (arrows) combined with meshed split-skin graft (arrowheads)

The inflammatory response improved, and his general condition recovered through intensive care, long-term cefazolin administration, aggressive rehabilitation, and appropriate nutritional management. However, the anterior chest abscess with sternum destruction was still infectious, and the left sciatic-rectal fossa abscess remained unresolved. To control the persistent infection, debridement of anterior chest wall, including the sternum, and sciatic-rectal fossa abscess incisional drainage were performed on the 24th day. The caudal half of the sternal body was resected, leaving the costal cartilage attachments in place (Fig. 3). Anterior chest wall incision was left open for postoperative daily washing. The patient’s general condition further improved without developing respiratory failure. Cefazolin was continued for 11 weeks. The patient was transferred to the general ward on the 43rd day. A latissimus dorsi myocutaneous flap was used to fill the sternal defect for wound closure and infection control 40 days after the second surgery when his general condition was further recovered (Fig. 3). The patient was finally discharged home, with anterior chest wall wound being closed, after 5 months of hospitalization.

## Discussion

SAB is one of the most common significant bacterial infections, with a recently estimated incidence of 25 per 100,000 population [3]. The Japanese national database survey reported approximately 14,400 adult cases and 700 pediatric cases in 2018 [4]. Deep organ infections are often associated with SAB. A French large multicentric prospective study demonstrated that the incidence of deep organ infection was 37%, and the most frequent was infective endocarditis (11%) and pneumonia (8%), followed by osteoarticular infections (6%) and urinary tract infections (5%) [2]. The patient had sustained an anterior chest wall injury 2 weeks before admission, which may have provided a route of entry for *S. aureus*, leading to SAB with a relatively rare mediastinal abscess.

Multi-stage surgical drainage is chronologically described in Table 1. The resection of the infectious sternum poses a clinical dilemma; while adequate and extensive debridement is desirable in terms of infection, sternal defects can cause postoperative respiratory failure and prolonged ventilator support. Autologous tissue, such as the pectoralis major or latissimus dorsi, is recommended for small-to-moderate defects. In contrast, alloplastic reconstruction is recommended for defects larger than 5 cm or involving four or more ribs [6]. In this case, a prosthetic replacement was contraindicated because of severe infection. Furthermore, as the patient was hypotrophic and in an unfavorable general condition, it was decided that the autologous muscle flap would most likely not be successful at the initial examination. The sternum was left intact in the first surgery, and sternum debridement alone without reconstruction was selected for the second surgery, followed by the latissimus dorsi myocutaneous flap after further improvement in his general condition. It would be essential to remove as much of the infective foci as possible, balancing the invasiveness of the procedures with the patient's general condition. We believe that this strategy contributed to the prevention of postoperative respiratory failure and early recovery of his general condition, even though a slight local infection persisted, and several small debridement were required even after the latissimus dorsi myocutaneous flap reconstruction.

**Table 1:** Time series of pathological conditions and surgical procedures

Time course	Surgical procedures to control pathological condition
1 day before admission	– Incisional drainage for cellulitis of the bilateral feet*
1st day (on admission)	– Percutaneous drainage for anterior mediastinal abscess
	– Incisional drainage for anterior chest wall abscess
2nd day	– Chest tube drainage for right pyothorax
	– Further incisional drainage for cellulitis of the bilateral feet
3rd day	• Debridement of anterior chest wall abscess
	• Thoracoscopic pleural curettage for right pyothorax
	• Debridement of the lower extremities, including amputation of right second and left fifth toe, for necrotizing fasciitis
24th day	• Incisional drainage for sciatic rectus fossa abscess
	• Debridement of the sternum for anterior chest wall infection
2 months	• Latissimus dorsi myocutaneous flap to fill the sternal defect
5 months	Discharged home

Bullets indicate surgical procedures under general anesthesia. Asterisk means that the procedure was performed at another hospital

## Conclusions

Repeated maximal removal of the infective foci with acceptable invasiveness resulted in the successful treatment of severe multiple deep organ abscesses, including a mediastinal abscess with sternum destruction, associated with SAB.

## Abbreviations

SAB: *Staphylococcus aureus* Bloodstream infection
MSSA: Methicillin-sensitive *Staphylococcus aureus*
CT: Computed tomography
WBC: White blood cell count
APACHEII: Acute Physiology and Chronic Health Disease Classification System II
ICU: Intensive care unit
